# Evaluation of sexual dysfunction and its predictors in men with obstructive sleep apnea syndrome: a multidimensional clinical and psychological approach

**DOI:** 10.3389/fneur.2026.1778854

**Published:** 2026-03-10

**Authors:** Mehmet Kabak, Barış Çil

**Affiliations:** 1 Department of Chest Diseases, Mardin Artuklu University, Mardin, Türkiye; 2 Department of Chest Diseases, Mardin Training and Research Hospital, Mardin, Türkiye

**Keywords:** anxiety, depression, erectile dysfunction, obstructive sleep apnea, sexual dysfunction

## Abstract

**Background:**

Obstructive sleep apnea syndrome (OSAS) is a common sleep-related breathing disorder associated with significant cardiovascular, metabolic, and neuropsychological consequences. Increasing evidence suggests that OSAS may also adversely affect male sexual function; however, sexual health remains underrecognized in routine clinical practice. This study aimed to evaluate the prevalence of sexual dysfunction in men with OSAS and to identify its clinical, psychological, and polysomnographic predictors using a multidimensional approach.

**Methods:**

This cross-sectional study included 52 men diagnosed with OSAS and 40 age-matched healthy controls. All participants underwent overnight type 1 polysomnography. Daytime sleepiness, anxiety, depression, sexual function, and quality of life were assessed using the Epworth Sleepiness Scale (ESS), Beck Anxiety Inventory (BAI), Beck Depression Inventory (BDI), Arizona Sexual Experience Scale (ASEX), and SF-36 questionnaire, respectively. Associations between sexual dysfunction and demographic, psychological, and sleep-related parameters were analyzed. Receiver operating characteristic (ROC) curve analysis was performed to evaluate predictive factors.

**Results:**

Sexual dysfunction was significantly more prevalent in men with OSAS compared to controls (*p* = 0.04) and was strongly associated with increasing OSAS severity. Men with sexual dysfunction exhibited significantly higher apnea–hypopnea index (AHI), ESS, anxiety, and depression scores, along with lower physical functioning, vitality, and mental health scores (all *p* < 0.05). ROC analysis demonstrated that AHI was a significant associated factor of sexual dysfunction (AUC = 0.709), with a sensitivity of 91% and specificity of 81% at a cutoff value of 59.75.

**Conclusion:**

Sexual dysfunction in men is closely related to the severity of OSAS rather than its mere presence. AHI emerges as a key associated factor, highlighting the importance of comprehensive sexual and psychological assessment in the clinical management of men with OSAS.

## Introduction

Obstructive sleep apnea syndrome (OSAS) is a prevalent sleep-related breathing disorder characterized by recurrent episodes of upper airway collapse during sleep, leading to intermittent hypoxia, increased sympathetic activation, and sleep fragmentation (1). It affects approximately 15–30% of men in the general adult population and is strongly associated with risk factors such as obesity, aging, and male sex (2, 3).

While OSAS is widely recognized for its contribution to cardiovascular diseases, insulin resistance, and neurocognitive dysfunctions, growing evidence highlights its impact on sexual health, particularly in men (4, 5). Male sexual dysfunction commonly manifesting as reduced libido, erectile dysfunction, and diminished sexual satisfaction has been observed at higher rates in patients with moderate to severe OSAS (6). Despite this, sexual health often remains under-assessed in sleep medicine clinics, partly due to social stigma, lack of awareness, and the multifactorial nature of sexual dysfunction.

The pathophysiology linking OSAS to sexual dysfunction is complex and multifactorial. Chronic nocturnal hypoxemia, altered hypothalamic–pituitary-gonadal axis function, endothelial dysfunction, and psychological distress (e.g., anxiety and depression) have all been implicated (7, 8). Moreover, hormonal disturbances such as low testosterone levels frequently observed in OSAS may further impair sexual performance (9).

Recent studies have employed objective assessments such as the Arizona Sexual Experience Scale (ASEX) and polysomnographic data to demonstrate a significant association between AHI (Apnea–Hypopnea Index) severity and sexual impairment (10, 11). However, the exact contribution of sleep-disordered breathing to sexual dysfunction remains underexplored and warrants further investigation, especially in male populations.

This study aims to examine the relationship between OSAS and sexual dysfunction in men by evaluating sleep parameters, anxiety and depression levels, quality of life, and sexual functioning scores. Understanding this relationship could aid in the development of more holistic approaches to the management of sleep apnea and improve patient outcomes in both medical and psychosocial domains.

## Materials and methods

This cross-sectional study was conducted between September 2024 and May 2025 after obtaining approval from the local ethics committee. During the study period, a total of 115 male patients were admitted to the sleep clinic. Based on polysomnographic evaluation, 65 patients had an AHI > 5 and were classified as having obstructive sleep apnea syndrome (OSAS), while 50 patients had an AHI < 5. After excluding individuals who declined to participate, 52 patients with OSAS and 40 healthy men without sleep-disordered breathing were ultimately included in the study. Demographic data including age, weight, height, and body mass index (BMI) were recorded. Analyses involving OSAS patients were performed to evaluate the association between disease severity and sexual dysfunction rather than to assess the presence of OSAS itself.

Several validated instruments were administered to all participants: Epworth Sleepiness Scale (ESS) for daytime sleepiness, Beck Anxiety Inventory (BAI), Beck Depression Inventory (BDI), Arizona Sexual Experience Scale (ASEX), SF-36 Quality of Life Questionnaire. In addition, all participants underwent routine blood tests, including thyroid function and hormone panels.

All participants completed an overnight type 1 polysomnography (PSG), which included 4-channel EEG (C4-A2, C3-A1, O2-A1, and O1-A2), bilateral electro-oculography (EOG), submental electromyography (EMG), nasal pressure monitoring via cannula, a thermistor to monitor airflow, anterior tibialis sensors, thoraco-abdominal belts to assess respiratory effort, electrocardiography (ECG), pulse oximetry, a neck microphone for snoring, and a chest sensor for body position. A minimum of 6 h of PSG data was required. Scoring was based on standard AASM (American Academy of Sleep Medicine) criteria. Apnea was defined as complete cessation of airflow for at least 10 s. Hypopnea was defined as partial cessation with ≥4% oxygen desaturation. AHI was calculated as the number of apnea and hypopnea events per hour of sleep and classified as: <5 (normal), 5–14 (mild), 15–29 (moderate), ≥30 (severe) (12).

The Epworth Sleepiness Scale assesses the likelihood of falling asleep during daily activities. It includes 8 items scored from 0 to 3, with higher scores indicating greater sleepiness (13).

The Beck Anxiety Inventory is a 21-item self-report measure assessing anxiety symptoms, each scored from 0 to 3 (14, 15). Total scores categorize anxiety as: 0–7 (minimal), 8–15 (mild), 16–25 (moderate), 26–63 (severe).

The Beck Depression Inventory consists of 21 items, also scored from 0 to 3, to assess depression severity (16, 17). Total score ranges: 0–9 (minimal), 10–16 (mild), 17–29 (moderate), 30–63 (severe).

The Arizona Sexual Experience Scale includes 5 items evaluating sexual drive, psychological and physiological arousal (penile erection), orgasmic capacity, and orgasm satisfaction (18, 19). Sexual dysfunction is defined as: a total ASEX score ≥19, or a score of ≥4 in ≥3 items, or a score of 5–6 in any one item.

The SF-36 questionnaire evaluates 8 dimensions of health-related quality of life over the past 4 weeks: physical functioning, social functioning, role limitations (physical and emotional), pain, general health, vitality, and mental health. Turkish validity and reliability were confirmed by Jafari et al. (20) and Koçyiğit et al. (21).

### Statistical analysis

All data were analyzed using SPSS version 26.0 (IBM Corp., Armonk, NY). Normality was assessed using the Shapiro–Wilk test. Between-group comparisons were performed using the Student’s *t*-test for parametric variables. Between group comparisons were performed using Ki–Kare test for nonparametric variables. One-way analysis of variance (ANOVA) was used to evaluate differences among the groups. ROC curve analysis was conducted for parameters showing statistical significance, and sensitivity, specificity, and AUC values were reported. A *p*-value of <0.05 was considered statistically significant.

## Results

The mean age was significantly higher in the group with sexual dysfunction (*p* = 0.047), suggesting that increasing age may negatively impact sexual health. Patients with sexual dysfunction had significantly higher daytime sleepiness scores (*p* = 0.003), indicating a potential link between sleep disturbances and impaired sexual function (Table 1).

**Table 1:** Comparison of sociodemographic, clinical, psychological, and sleep parameters between men with and without sexual dysfunction.

Variable	Sexual dysfunction (Mean ± SD) (*n* = 24)	No sexual dysfunction (Mean ± SD) (*n* = 68)	*p*-value
Age (years)	48.042 ± 9.15	43.662 ± 9.19	0.047
BMI (kg/m^2^)	30.450 ± 4.98	28.441 ± 4.46	0.069
Weight (kg)	92.583 ± 15.61	88.000 ± 17.12	0.252
Height (cm)	174.375 ± 7.37	173.618 ± 11.88	0.771
Epworth sleepiness scale	7.583 ± 4.32	4.426 ± 4.35	0.003
Physical functioning	55.625 ± 28.30	73.382 ± 25.27	0.005
Role limitations (physical)	64.583 ± 39.64	69.853 ± 37.55	0.562
Pain	59.479 ± 26.82	72.463 ± 24.45	0.032
General health perception	43.958 ± 20.64	60.074 ± 20.08	0.001
Vitality	47.708 ± 17.51	65.221 ± 20.21	<0.001
Social functioning	65.104 ± 23.31	75.956 ± 22.86	0.050
Mental health	55.958 ± 18.33	66.824 ± 17.81	0.012
Beck anxiety inventory	16.042 ± 8.65	10.221 ± 9.90	0.012
Beck depression inventory	14.750 ± 11.66	9.750 ± 8.19	0.024
Arizona sexual experience score	17.42 ± 2.06	10.49 ± 2.66	<0.001
Apnea-hypopnea index (AHI)	50.268 ± 28.07	30.952 ± 19.53	0.002
Estradiol (pg/mL)	22.814 ± 7.19	28.131 ± 11.91	0.043
Vitamin D (ng/mL)	14.275 ± 6.26	14.583 ± 5.65	0.824

Physical function scores were significantly lower in those with sexual dysfunction (*p* = 0.005), highlighting the role of general physical health in sexual performance. The sexual dysfunction group reported significantly higher pain levels (*p* = 0.032), suggesting that chronic pain may adversely affect sexual activity. Participants with sexual dysfunction had poorer self-reported general health (*p* = 0.001), indicating a possible positive correlation between perceived general health and sexual well-being. Energy levels were significantly lower among individuals with sexual dysfunction (*p* < 0.001). Fatigue and lack of vitality may contribute to reduced sexual desire. Social functioning scores were lower in the sexual dysfunction group (*p* = 0.050), implying that sexual problems may negatively influence social interactions and quality of life. Mental health scores were significantly lower in the sexual dysfunction group (*p* = 0.012), underscoring the potential impact of psychological well-being on sexual functioning (Table 1).

Levels of both anxiety (*p* = 0.012) and depression (*p* = 0.024) were significantly higher among men with sexual dysfunction, indicating strong psychological comorbidity (Table 1).

As expected, ASEX scores were markedly higher in the sexual dysfunction group (*p* < 0.001), confirming the diagnostic validity of the classification (Table 1).

The group with sexual dysfunction had significantly higher AHI scores (*p* = 0.002), supporting the hypothesis that poor sleep quality due to obstructive sleep apnea may impair sexual function (Table 1).

The prevalence of sexual dysfunction was significantly higher in patients with AHI > 59.75 (*p* = 0.01), suggesting a correlation between apnea severity and impaired sexual function. No statistically significant relationship was found between minimum oxygen saturation and sexual dysfunction (*p* = 0.40) (Table 2).

**Table 2:** Analysis of categorical variables related to sexual dysfunction.

Variable	Subgroup	No sexual dysfunction (*n* = 68)	Sexual dysfunction (*n* = 24)	Total	*p*-value
AHI	<59.75	54 (79.4%)	11 (45.8%)	65	0.01
	>59.75	14 (20.6%)	13 (54.2%)	27	
OSAS	Absent	25 (36.7%)	3 (12.5%)	28	0.026
	Present	43 (63.3%)	21 (87.5.9%)	64	
Minimum SO₂	<74.50	19 (27.9%)	5 (20.8%)	24	0.40
	>74.50	49 (72.1%)	19 (79.2%)	68	
Beck anxiety inventory	No anxiety	35 (51.4%)	3 (12.5%)	38	0.09
	Mild anxiety	15 (22%)	11 (45.9%)	26	
	Moderate anxiety	10 (14.7%)	6 (25%)	16	
	Severe anxiety	8 (11.9%)	4 (16.6%)	12	
Beck depression inventory	Minimal depression	37 (54.4%)	8 (33.3%)	45	0.07
	Mild depression	19 (27.9%)	8 (33.3%)	27	
	Moderate depression	11 (16.1%)	5 (20.8.7%)	16	
	Severe depression	1 (1.6%)	3 (12.4%)	4	
OSAS severity	No OSAS	25 (36.8%)	3 (12.5%)	28	0.02
	Mild OSAS	7 (10.2%)	3 (12.5%)	10	
	Moderate OSAS	16 (23.5%)	3 (12.5%)	19	
	Severe OSAS	20 (29.5%)	15 (62.5%)	35	

Sexual dysfunction was more frequent among patients compared to controls (*p* = 0.026), indicating the possible impact of disease status on sexual health. Men with OSAS had significantly higher rates of sexual dysfunction than those without, likely due to reduced oxygenation and sleep fragmentation Severe OSAS was strongly associated with sexual dysfunction (62.5% prevalence), significantly more than those without OSAS (12.5%) (*p* = 0.02) (Table 2).

Higher anxiety levels were associated with greater prevalence of sexual dysfunction. In the severe anxiety group, over 16.6% had dysfunction (*p* = 0.009). Sexual dysfunction decreased with the severity of depression. In the severe depression group, 12.4% had sexual dysfunction (*p* = 0.07) (Table 2).

For the BDI, patients with moderate OSAS demonstrated significantly higher depression scores compared to individuals without OSAS (mean difference = 0.71, *p* = 0.028). No significant differences were observed between the no OSAS group and patients with mild or severe OSAS, nor among the OSAS severity subgroups themselves (Table 3).

**Table 3:** Multiple comparisons.

Dependent variable			Mean difference (I-J)	Std. error	Sig.	95% Confidence Interval	
						Lower bound	Upper bound
Beck depression inventory	No OSAS	Mild OSAS	−0.6071	0.3539	0.514	−1.731	0.516
		Moderate OSAS	−0.7124^*^	0.2333	0.028	−1.369	−0.056
		Sever OSAS	−0.4357	0.2004	0.186	−0.981	0.110
	Mild OSAS	No OSAS	0.6071	0.3539	0.514	−0.516	1.731
		Moderate OSAS	−0.1053	0.3891	1.000	−1.275	1.065
		Sever OSAS	0.1714	0.3703	0.998	−0.968	1.310
	Moderate OSAS	No OSAS	0.7124^*^	0.2333	0.028	0.056	1.369
		Mild OSAS	0.1053	0.3891	1.000	−1.065	1.275
		Sever OSAS	0.2767	0.2576	0.871	−0.436	0.990
	Sever OSAS	No OSAS	0.4357	0.2004	0.186	−0.110	0.981
		Mild OSAS	−0.1714	0.3703	0.998	−1.310	0.968
		Moderate OSAS	−0.2767	0.2576	0.871	−0.990	0.436
Beck anxiety inventory	No OSAS	Mild OSAS	−0.8357	0.3314	0.142	−1.859	0.188
		Moderate OSAS	−1.0620^*^	0.3130	0.013	−1.950	−0.174
		Sever OSAS	−0.6500^*^	0.2266	0.034	−1.266	−0.034
	Mild OSAS	No OSAS	0.8357	0.3314	0.142	−0.188	1.859
		Moderate OSAS	−0.2263	0.4101	0.995	−1.407	0.954
		Sever OSAS	0.1857	0.3486	0.996	−0.860	1.232
	Moderate OSAS	No OSAS	1.0620^*^	0.3130	0.013	0.174	1.950
		Mild OSAS	0.2263	0.4101	0.995	−0.954	1.407
		Sever OSAS	0.4120	0.3312	0.779	−0.516	1.340
	Sever OSAS	No OSAS	0.6500^*^	0.2266	0.034	0.034	1.266
		Mild OSAS	−0.1857	0.3486	0.996	−1.232	0.860
		Moderate OSAS	−0.4120	0.3312	0.779	−1.340	0.516
Arizona sexual experiences scale	No OSAS	Mild OSAS	−0.22857	0.16059	0.702	−0.7423	0.2852
		Moderate OSAS	−0.08647	0.09921	0.949	−0.3660	0.1931
		Sever OSAS	−0.38571^*^	0.09877	0.002	−0.6556	−0.1158
	Mild OSAS	No OSAS	0.22857	0.16059	0.702	−0.2852	0.7423
		Moderate OSAS	0.14211	0.17527	0.966	−0.3890	0.6732
		Sever OSAS	−0.15714	0.17502	0.945	−0.6861	0.3718
	Moderate OSAS	No OSAS	0.08647	0.09921	0.949	−0.1931	0.3660
		Mild OSAS	−0.14211	0.17527	0.966	−0.6732	0.3890
		Sever OSAS	−0.29925	0.12119	0.099	−0.6321	0.0336
	Sever OSAS	No OSAS	0.38571^*^	0.09877	0.002	0.1158	0.6556
		Mild OSAS	0.15714	0.17502	0.945	−0.3718	0.6861
		Moderate OSAS	0.29925	0.12119	0.099	−0.0336	0.6321

*The mean difference is significant at the 0.05 level.

Regarding the BAI, anxiety scores were significantly higher in patients with moderate OSAS and severe OSAS compared to the no OSAS group (mean differences = 1.06 and 0.65, respectively; *p* = 0.013 and *p* = 0.034). Comparisons involving mild OSAS did not reach statistical significance (Table 3).

For the ASEX, a significant difference was observed only between patients with severe OSAS and those without OSAS, with higher ASEX scores indicating greater sexual dysfunction in the severe OSAS group (mean difference = 0.39, *p* = 0.002). No significant differences were found between mild or moderate OSAS groups and controls, nor between mild and moderate OSAS (Table 3).

**Figure 1:**
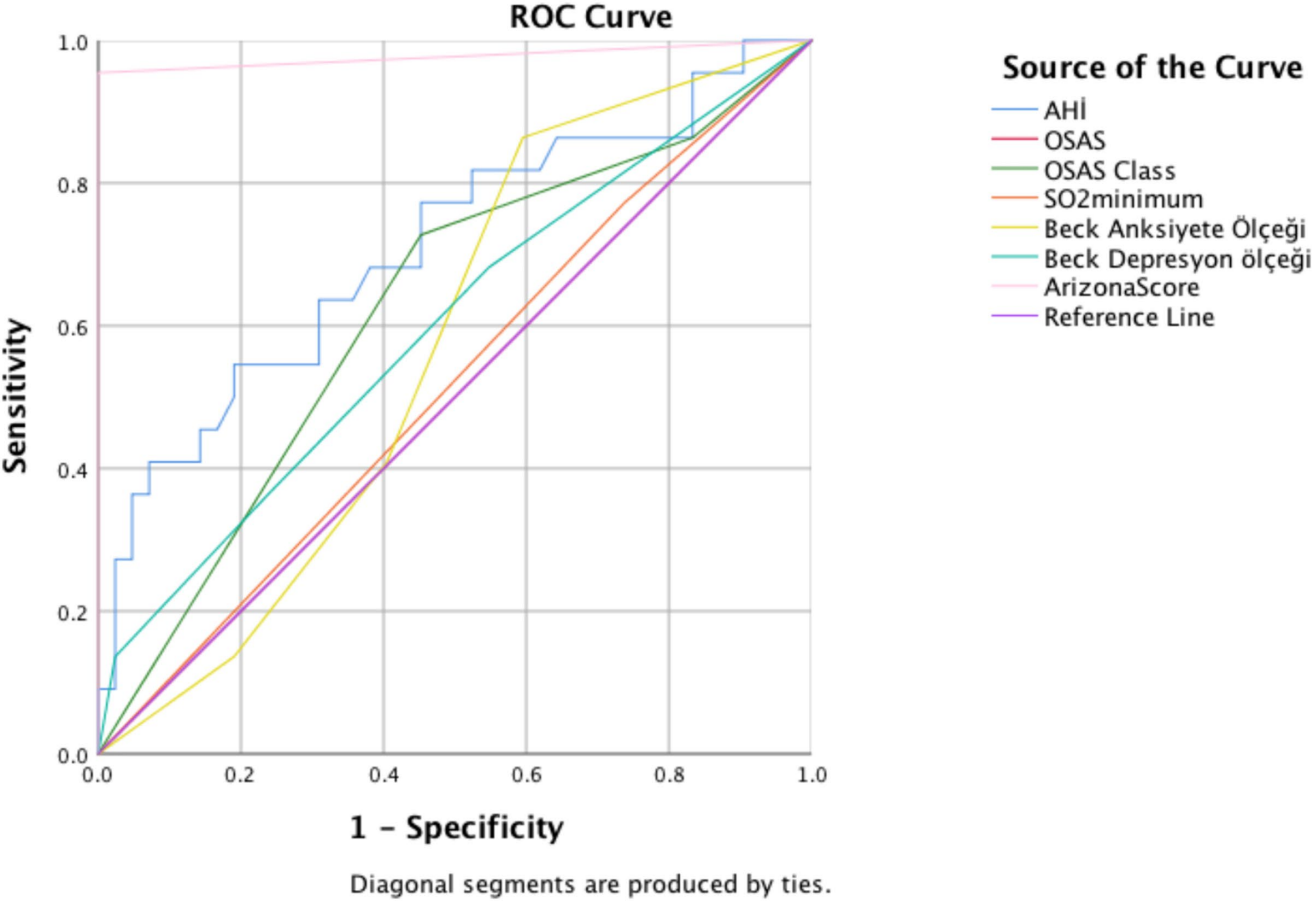
In the ROC curve analysis, the area under curve of the Arizona score was calculated as 0.977, *p* < 0.001, the area under curve of the Apnea Hypopnea Index was 0.709, *p* = 0.006, the area under curve of the beck anxiety score was 0.362, *p* = 0.072 the area under curve of the beck depression score was 0.597, *p* = 0.203, and the area under curve of the So2 minimum was 0.517, *p* = 0.821.

ROC curve analysis was performed for significant parameters. AHI and Arizona score were found to be significantly higher in area under curve (Figure 1). The sensitivity 91% and specificity 81% of AHI were measured (Table 4).

**Table 4:** Sensitivity and specificity of parameters affecting sexual dysfunction.

Parameter	Cut-off	Sensitivity	Specificity
Apnea hypopnea index (AHI)	59,75	91	81
Arizona score	15,5	98	95

## Discussion

This study investigated the relationship between OSAS and sexual dysfunction in men, focusing on physiological, psychological, and quality-of-life factors. The results revealed that men with sexual dysfunction exhibited significantly higher levels of daytime sleepiness, anxiety, depression, and apnea severity, alongside lower physical, mental, and general health scores.

The significant association between age and sexual dysfunction aligns with the known age-related decline in testosterone levels, endothelial function, and sexual performance in men (22). Additionally, excessive daytime sleepiness, as assessed by the ESS, was significantly higher in the sexual dysfunction group, indicating that sleep fragmentation and fatigue may contribute to reduced libido and sexual satisfaction (23, 24). These findings are supported by previous evidence showing that excessive daytime sleepiness, as measured by the Epworth Sleepiness Scale, significantly contributes to psychological distress, thereby reinforcing the relevance of ESS-related findings in the interpretation of our results (25).

In the psychological domain, both Beck Anxiety and Depression Inventories demonstrated significantly higher scores among men with sexual dysfunction. These findings are consistent with previous research suggesting that mental health disorders such as anxiety and depression are common comorbidities of both OSAS and sexual dysfunction, potentially exacerbated by chronic sleep disruption and perceived loss of health (26, 27).

Quality-of-life assessments using the SF-36 revealed that men with sexual dysfunction scored significantly lower in multiple domains, including physical functioning, vitality, general health perception, and mental health. This reflects the multidimensional burden of OSAS and suggests that sexual dysfunction may be both a consequence and a contributor to reduced quality of life in affected individuals (28).

Apnea–hypopnea index, a measure of OSAS severity, was significantly higher in the sexual dysfunction group and showed the highest area under the curve in ROC analysis. This suggests that AHI may serve as a moderately accurate associated factor of sexual dysfunction. In contrast, the presence of OSAS alone and minimum oxygen saturation lacked discriminative power. These findings suggest that the severity rather than the presence of OSAS is more relevant to sexual outcomes, supporting previous studies linking severe OSAS with erectile dysfunction and altered hormonal profiles (29, 30).

The present study demonstrated that increasing OSAS severity, along with higher levels of depression and anxiety and elevated AHI values, was significantly associated with greater sexual dysfunction. These findings are in line with previous evidence suggesting that sexual impairment in OSAS arises from a complex interaction between physiological and psychological factors (31). Chronic intermittent hypoxia and sleep fragmentation characteristic of OSAS contribute to endothelial dysfunction, reduced nitric oxide bioavailability, and hemodynamic instability, all of which play a central role in erectile and sexual function. Furthermore, the frequent coexistence of metabolic syndrome may exacerbate vascular and hormonal dysregulation, thereby amplifying sexual dysfunction (32, 33).

Recent literature has emphasized the multidimensional nature of the relationship between OSAS and sexual dysfunction, indicating that apnea severity alone does not fully explain sexual impairment. Psychological burden, impaired sleep quality, and systemic inflammation have also been identified as important contributing factors (34). In this context, our findings add to the growing body of evidence by showing that sexual dysfunction becomes particularly pronounced in patients with more severe OSAS, underscoring the clinical relevance of disease severity. Notably, while OSAS severity categories demonstrated a modest ability to discriminate sexual dysfunction, psychological measures such as anxiety and depression scales showed limited independent discriminatory performance. This suggests that although psychological distress is clinically relevant in men with OSAS, it may not be sufficient as a standalone screening indicator for sexual dysfunction and should be interpreted in conjunction with objective disease severity measures.

Recent evidence suggests that OSAS-targeted therapies can influence sexual function outcomes in affected patients. A systematic review and meta-analysis reported that continuous positive airway pressure (CPAP) therapy significantly improved erectile function and sexual parameters in most studies of OSA patients with erectile dysfunction, although sildenafil demonstrated a greater therapeutic impact when used alone; combined CPAP and PDE5 inhibitor treatment may offer cumulative benefits for sexual function in this population sildenafil had a more substantial effect on IIEF-5 scores than CPAP alone, but both treatments were associated with improvements in sexual outcomes (35, 36). Therefore, future studies should focus on integrated treatment approaches addressing both sleep disordered breathing and sexual dysfunction to optimize clinical outcomes in this patient population.

This study has several limitations that should be acknowledged. First, the sample size was relatively small and limited to a single center, which may reduce the generalizability of the findings. Second, the cross-sectional design precludes any causal inference between OSAS severity and sexual dysfunction. Third, the study relied on self-reported scales such as ASEX, BAI, and BDI, which may be subject to response bias or underreporting, especially in sensitive areas like sexual health. Additionally, hormonal levels such as testosterone were not comprehensively assessed, which limits our ability to evaluate potential endocrine contributions to sexual dysfunction. Finally, unmeasured confounding factors such as medication use, comorbidities, or marital relationship quality may have influenced the outcomes.

## Conclusion

This study demonstrates that sexual dysfunction in men is significantly associated with the severity of obstructive sleep apnea, rather than its mere presence. AHI showed a relatively stronger association compared to other variables; however, its ability to discriminate outcomes should be interpreted with caution given the cross-sectional nature of the study. Additionally, elevated levels of anxiety and depression, along with decreased quality-of-life indicators such as vitality and physical functioning, were more prevalent among men with sexual dysfunction.

## Data Availability

The original contributions presented in the study are included in the article/supplementary material, further inquiries can be directed to the corresponding author.
